# Mesenchymal stem cell-based therapy in kidney transplantation

**DOI:** 10.1186/s13287-016-0283-6

**Published:** 2016-02-07

**Authors:** Cheng Chen, Jianquan Hou

**Affiliations:** Department of Urology, The First Affiliated Hospital of Soochow University, 188 Shizi Road, Suzhou, 215006 Jiangsu PR China

## Abstract

Kidney transplantation is the best treatment for end-stage renal disease, but its implementation is limited by organ shortage and immune rejection. Side effects of current immunosuppressive drugs, such as nephrotoxicity, opportunistic infection, and tumorigenic potential, influence long-term graft outcomes. In recent years, continued research and subsequent discoveries concerning the properties and potential utilization of mesenchymal stem cells (MSCs) have aroused considerable interest and expectations. Biological characteristics of MSCs, including multi-lineage differentiation, homing potential, paracrine effect and immunomodulation, have opened new horizons for applications in kidney transplantation. However, many studies have shown that the biological activity of MSCs depends on internal inflammatory conditions, and the safety and efficacy of the clinical application of MSCs remain controversial. This review summarizes the findings of a large number of studies and aims to provide an objective viewpoint based on a comprehensive analysis of the presently established benefits and obstacles of implementing MSC-based therapy in kidney transplantation, and to promote its clinical translation.

## Background

The number of patients with chronic kidney disease and end-stage renal disease (ESRD) increases each year. Kidney transplantation is an effective long-term means of treatment for ESRD, but is closely accompanied by a high risk of post-transplant rejection. With improvements in tissue type matching and the use of new immunosuppressive agents, the danger of acute renal post-transplant rejection has been greatly reduced, but the hazards for long-term graft survival have remained unchanged.

Regular immunosuppressive drug protocols mainly include corticosteroids in combination with calcineurin inhibitors, purine/pyrimidine synthesis inhibitors, and sometimes lymphodepletion [1]. However, their use should be gradually tapered off to lower the risk of opportunistic infections and malignancies, as well as to prevent long-term toxicity to the central nervous, gastrointestinal and hematopoietic systems and kidneys. Therefore, there is a substantial need for novel immunosuppressive therapies that have few side effects, while maintaining efficacy.

Mesenchymal stem cells (MSCs) are mesoderm-derived multipotent stromal cells that have high self-renewal and multi-lineage differentiation potential, an immunomodulatory and inflammatory homing capacity, and the ability to repair damaged tissues and organs. They are widespread in many tissues of the body and can be cultured and multiplied in vitro, with the ability to differentiate into bone, cartilage, fat, muscle, nerves and other tissues under certain conditions [2, 3].

The underlying immunomodulatory mechanism of action of MSCs has been widely recognized, and its effectiveness has also been confirmed by early clinical trials in the treatment of graft-versus-host disease (GVHD) [4]. Thus, we can speculate that the application of MSCs in kidney transplantation will not only reduce the amount of immunosuppressive drugs required, but also enhance the renal function of patients and improve their long-term survival.

## Biological characteristics of MSCs

MSCs are pluripotent, long, shuttle-shaped, fibroblast-like stem cells that are present in almost all tissues and organs, including the bone marrow, periosteum, fat tissue, pulp, synovium, umbilical cord, placenta, amniotic fluid and fetal tissues.

To date, no studies have reported the discovery of specific MSC surface markers. In view of this, in 2006, the International Society of Cellular Therapy defined MSCs by the following three minimum criteria [5]: (1) MSCs must adhere to plastic under standard tissue culture conditions; (2) MSCs must express specific cell surface markers such as CD73, CD90 and CD105, while lacking the expression of hematopoietic stem cell markers and autologous transplant rejection antigens such as CD45, CD34, CD14, CD11b, CD79α, CD19 and human leukocyte antigen (HLA)-DR; (3) in vitro, these cells must have the capacity to be induced to differentiate into adipocytes, osteoblasts and chondrocytes.

MSCs have multi-lineage differentiation potential; in vitro, they can differentiate into mesoderm lineage (bone, cartilage, fat, and connective tissues) and transdifferentiate into both ectodermal (nerve and epithelial tissues) and endodermal cells (muscle and liver cells) [2, 3]. Thus, MSCs have become the preferred seed cells for tissue engineering.

After systemic infusion, MSCs can preferentially migrate into sites of inflammation and injury and tumors. This ‘homing capacity’ of MSCs can be used in drug or gene carrier applications for targeted treatment of diseases or killing of tumor cells [6]. In the situation of renal ischemia-reperfusion injury, MSCs move towards renal tissues and repair ischemic renal tubular injury. Through paracrine and endocrine secretion of various cytokines, MSCs inhibit apoptosis and fibrosis of damaged renal tissues, regulate immune and inflammatory responses, and promote angiogenesis to stimulate renal tissue regeneration instead of directly differentiating into renal tubular epithelial cells [7].

## Plasticity of the immunoregulatory and anti-inflammatory properties of MSCs

MSCs constitutively express low levels of major histocompatibility complex (MHC) class I molecules, but do not express MHC class II and costimulatory molecules, including B7-1 (CD80), B7-2 (CD86) and CD40 [8], resulting in the absence of a HLA allogeneic barrier during their transplantation. Thus, the lack of immunogenicity of MSCs provides a theoretical basis for their use in extensive transplantation, such that a cord blood bank, for example, could be established.

MSC-mediated immunosuppression is elicited by interferon (IFN)-γ in the presence of other proinflammatory cytokines, tumor necrosis factor-α, interleukin (IL)-1α, or IL-1β [9, 10]. This activation step has also been shown in vivo in a GVHD model, since recipients of IFN^−/−^ T cells did not respond to MSC treatment and succumbed to GVHD [11]. The immunosuppression of MSCs was absent in IFN-γ receptor 1^−/−^ mice, highlighting the importance of IFN-γ in this process [10].

**Fig. 1:**
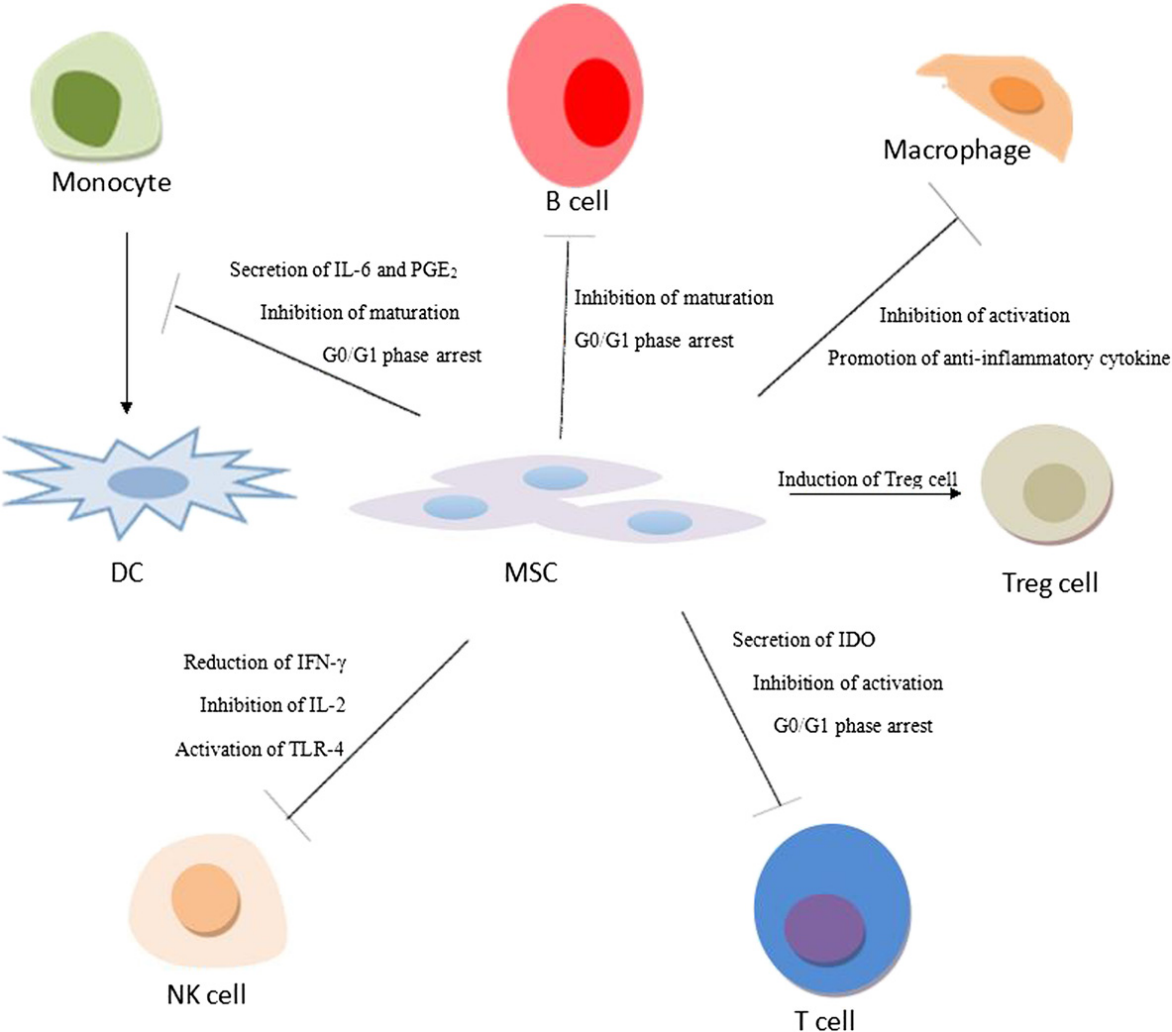
Immunomodulatory effects of mesenchymal stem cells (*MSCs*) on immune cells. MSCs inhibit the activation of macrophages and proliferation of T cells, regulate the activation and differentiation of B cells, and suppress natural killer (*NK*) cell proliferation. They inhibit monocyte differentiation into dendritic cells (*DC*) and enhance the cell function of CD^+^Cd25^+^FoxP3^+^ T regulatory (*Treg*) cells. They secrete inhibitory factors or make cell-to-cell contacts with various types of immune cells to exert their immunosuppressive effects and treat a variety of immune system disorders. *IDO* indoleamine 2,3-dioxygenase, *IFN* interferon, *IL* interleukin, *PGE*_*2*_ prostaglandin E_2_, *TLR* Toll-like receptor

MSCs exert immunosuppressive effects by secreting bioactive molecules and through cell-to-cell contact (Fig. 1). They can inhibit the activation of macrophages to release proinflammatory cytokines and promote anti-inflammatory cytokine secretion. By secreting the soluble antigen IL-6 and prostaglandin E_2_, MSCs suppress differentiation of monocytes into, and interfere with the development, differentiation and maturation of, dendritic cells. Furthermore, MSCs can reduce the secretion of IFN-γ in natural killer cells and change their phenotype, inhibiting their proliferation, cytokine secretion and cell killing role by the activation of Toll-like receptor (TLR)4 [12]. Together with IFN-γ stimulation, indoleamine 2,3-dioxygenase (IDO) secreted by MSCs can suppress T-cell proliferation by degrading tryptophan, an essential amino acid for the induction of the T-cell cycle, leading to arrest in G0/G1 phase. MSCs increase CD4^+^ CD25^+^ FoxP3^+^ T regulatory cell function and can cause the arrest of B lymphocytes in the G0/G1 phase [13]. Mediated by T cells, MSCs inhibit the maturation, migration, proliferation and antibody production of B cells [14].

MSC immunosuppressive functions are ‘licensed’ by the inflammation environment [10], and this inflammatory status determines the immunoregulatory effect of MSCs. Strong inflammation causes MSCs to suppress the immune response, whereas weak inflammation leads to immune reaction enhancement by MSCs. MSC1 and MSC2 represent the proinflammatory and anti-inflammatory phenotypes of MSCs, respectively [15]. The activation of TLRs can affect the inflammatory functions of MSCs [16]. In the absence of proinflammatory cytokines, the activation of TLR4 can result in the differentiation of MSCs into the MSC1 phenotype. Conversely, differentiation into the MSC2 phenotype can be induced by the delivery of anti-inflammatory signals to MSCs through TLR3 [15, 17].

## MSCs in kidney transplantation

Since MSCs can exert both anti-ischemia-reperfusion injury and anti-post-transplant rejection functions, these cells are of particular interest in kidney transplantation. Preclinically, MSC therapy positively influences renal function and graft survival [18, 19]. Since the first successful clinical application of MSCs in 2004 [4], research into MSC therapeutic applications has expanded to the point where there are now more than 500 registered clinical trials (http://clinicaltrials.gov/). However, only nine MSC clinical trials in kidney transplantation have been registered (Table 1). Despite this limited number, translation of MSCs from the bench to bedside for use in kidney transplantation is highly possible.

**Table 1:** Registered clinical trials of mesenchymal stem cells in kidney transplantation (ClinicalTrial.gov, updated July 2015)

NCT	Status	Title	Site	Type of MSC	Start date
NCT02409940	Recruiting	To elucidate the effect of mesenchymal stem cells on the T-cell repertoire of kidney transplant patients	Chandigarh, India	Autologous/allogeneic; BM-MSC	September 2013
NCT02387151	Recruiting	Allogeneic mesenchymal stromal cell therapy in renal transplant recipients	Leiden, Netherlands	Allogeneic; BM-MSC	March 2015
NCT02057965	Recruiting	Mesenchymal stromal cell therapy in renal recipients	Leiden, Netherlands	Autologous; BM-MSC	March 2014
NCT02012153	Recruiting	Mesenchymal stromal cells in kidney transplant recipients	Bergamo, Italy	Autologous; BM-MSC	December 2013
NCT00659620	Unknown	Mesenchymal stem cell transplantation in the treatment of chronic allograft nephropathy	Fuzhou, Fujian	Autologous; BM-MSC	May 2008
NCT00734396	Completed	Mesenchymal stem cells and subclinical rejection	Leiden, Netherlands	Autologous; BM-MSC	February 2009
NCT00752479	Terminated	Mesenchymal stem cells under basiliximab/low dose RATG to induce renal transplant tolerance	Bergamo, Italy	Autologous; BM-MSC	May 2008
NCT00658073	Completed	Induction therapy with autologous mesenchymal stem cells for kidney allografts	Fuzhou, Fujian	Autologous; BM-MSC	March 2008
NCT01429038	Recruiting	Mesenchymal stem cells after renal or liver transplantation	Liege, Belgium	Allogeneic; BM-MSC	February 2012

*BM-MSC* bone marrow-derived mesenchymal stem cell, *MSC* mesenchymal stem cell, *NCT* ClinicalTrials.gov identifier, *RATG* rabbit antithymocyte globulin

Reinders et al. [20] have demonstrated that combined treatment with autologous bone marrow-derived MSCs (BM-MSCs) and a maintenance immunosuppressant alleviates acute rejection and interstitial fibrosis/tubular atrophy up to 24 weeks after MSC infusion. Other findings have shown that autologous MSCs injected together with a maintenance immunosuppressant resulted in more stable graft function 1 year after transplantation [21]. Peng et al. [22] described a significant reduction of tacrolimus dose accompanied by stable renal function after allogeneic MSC application. Crop et al. [23] suggested that MSCs can inhibit the proliferation of T cells and reduce graft inflammation, indicating that MSC administration can be a promising cell therapy in clinical organ transplantation. Vanikar et al. [24] provided evidence that adipose-derived mesenchymal stem cells (ADMSCs) combined with hematopoietic stem cell transplantation perform more effectively than transplantation alone. Results from studies by Trivedi et al. [25] and Vanikar et al. [26] have shown that pre-transplant ADMSCs combined with hematopoietic stem cells minimized the overall application of immunosuppressants in kidney transplantation. Tan et al. [27] confirmed that inoculation with MSCs significantly reduced acute rejection incidence and opportunistic infections, while maintaining renal function better than that of controls. Donor MSC injection into the iliac bone at the time of kidney transplantation was reported to be safe [28]. A large-sample meta-analysis has also confirmed the clinical safety of MSC therapies [29].

## Problems in the clinical translation of MSCs

Although MSCs exhibit great potential in most preclinical and clinical data, many questions remain to be resolved. Furthermore, the optimal source of MSCs, the optimal time window, dosage, route and frequency of MSC administration, post-transplantation safety and long-term prognosis have still not been determined.

### MSC sources

MSCs are found in almost all postnatal organs and tissues, including fat, bone, cartilage, umbilical cord, cord blood, synovium, synovial fluid, muscle, skin and pulp. MSCs from different sources possess similar morphologies and express identical surface markers, but they do show differences in some biological characteristics. BM-MSCs are an important part of the bone marrow microenvironment, which promotes the function of hematopoietic stem cells through the secretion of IL-1a, IL-1b, IL-6, IL-7, IL-8, IL-11, IL-14, IL-15, leukemia inhibitory factor, stem cell factor, thrombopoietin, fetal liver tyrosine kinase (Flt)-3 ligand, macrophage colony-stimulating factor, granulocyte colony-stimulating factor, and granulocyte-macrophage colony-stimulating factor [30]. Umbilical cord blood mesenchymal stem cells are the youngest MSCs, with the highest potential and lowest immunogenicity [31, 32]. Human umbilical cord MSCs are mainly isolated from Wharton’s jelly in the umbilical cord, which can be easily, noninvasively and painlessly obtained without causing adverse effects on the fetus and mother, can be utilized without ethical restrictions, and does not produce teratomas [33]. ADMSCs have many sources, and these cells are easy to isolate and can be repeatedly collected with less pain to the donor. Human amniotic MSCs can be derived from abandoned amniotic membranes and possess low immunogenicity and multi-lineage differentiation, as well as anti-inflammatory abilities similar to those of BM-MSCs. The most promising candidate source of MSCs for cell therapy continues to be debated.

### Autologous or allogeneic MSCs

Thus far, most studies have used autologous cells. Togel et al. [34] reported that autologous MSCs were more potent than allogenic MSCs in a rat acute renal failure model. However, it takes several weeks to months to manufacture autologous cells due to the expansion period, quality controls and logistics, and this period of time is too long for patients in need of treatment. Moreover, MSCs derived from patients with renal disease have a lower capacity for kidney regeneration [35].

Allogeneic MSCs offer an ‘off the shelf’ advantage for clinical use and have the potential to be mass-produced rapidly [36]. This would significantly decrease costs and the number of procedures, and enable use of cells from young healthy donors that may exhibit higher efficacy than cells from older individuals.

### Timing of administration of MSCs

In a mouse model of GVHD, MSCs were most effective when administered at 3, 8, or 20 days after bone marrow transplantation, and these cells had no protective effect when applied on the day of transplantation, suggesting that a certain proinflammatory environment is necessary for MSCs to polarize into an anti-inflammatory phenotype [11, 17]. In contrast, Casiraghi et al. [37] assumed that better immune suppression by MSCs can be achieved when they are injected before transplantation rather than after it. Perico et al. [21, 38] also reported a decrease in kidney function when MSCs were administered within a week after transplantation. Therefore, the optimal timing for MSC administration remains under discussion.

### Route of administration of MSCs

To date, most trials in kidney transplantation have used intravenous infusion of MSCs. However, most of the cells accumulated in the lungs after administration and only a small number of cells reached the transplanted kidney, implying that most MSCs do not play a substantial role in the target tissue. Lung entrapment may be due to the small capillary size, large capillary network, and strong adhesion properties of MSCs. Increasing the number of administered MSCs to compensate for this disadvantage may cause unwanted side effects such as embolism [39]. Zonta et al. [40] reported that local arterial administration is more effective than systemic intravenous administration, while not causing arterial thrombosis, infections, or other adverse reactions.

### Dose of MSCs

The application of MSCs in a dose of 0.4–10 × 10^6^ cells per kilogram of body weight in humans is reportedly the most appropriate, since this dose does not result in significant adverse effects. However, a high-dose (450 × 10^6^ cells) infusion of MSCs in adult sheep was well tolerated and proven safe [41].

### Frequency of MSC administration

No long-lasting response was observed in children with steroid-resistant GVHD who received one infusion of MSCs, whereas two or more infusions led to better results [42]. However, Franquesa et al. [43] reported that a single delayed MSC injection was effective for the long-term protection of kidney allografts. Therefore, the most effective number and frequency of MSC infusions are uncertain and still to be determined.

### Diversity in the mechanisms of action of MSCs derived from different species

Murine MSCs exert immunosuppressive effects through nitric oxide generated by inducible nitric oxide synthase, whereas human MSCs use IDO as a major effector molecule. To eliminate species disparities that cause variations in different studies, an IDO-expressing humanized MSC mouse model has been created to mimic the human system [44].

### Synergistic role of MSCs with immunosuppressive drugs

Interestingly, MSCs perform better under the conditions of an immunosuppressant-resistant acute inflammatory disease. Le Blanc et al. [4] reported the treatment of severe steroid- and cyclosporine-resistant GVHD with MSCs. Sun et al. [45] described the profound therapeutic effect of human umbilical cord MSCs in severe and refractory systemic lupus erythematosus. Dalal et al. [46] found that patients with severe, non-drug-responsive Crohn’s disease can be treated with MSCs. However, the treatment of GVHD with a combination therapy of Prochymal (an MSC-based product) and steroids had no therapeutic effect [47]. The combined administration of MSCs with low-dose cyclosporine A (CsA) reversed the protective influence of CsA and accelerated allograft rejection [48]. Zhang et al. [49] found that, when combined with low-dose CsA, MSCs could not prolong animal survival compared with CsA monotherapy. Therefore, the application of MSCs is most feasible in the therapy of immunosuppressant-resistant patients.

### Other potential risks of MSCs

Other adverse events reported after MSC transplantation include fever, fungal infection, organ system complications (neurological, pulmonary, cardiovascular, gastrointestinal and renal, and hematologic), high recurrence rate, tumor growth and metastasis, and even death [29].

MSCs comprise a rare population of 0.001–0.01 % of all nucleated cells in the bone marrow, so a sufficient number of MSCs cannot be produced in 30 % of patients with recurrent bone marrow biopsy [50]. Moreover, large numbers of MSCs have to be expanded in vitro to obtain amounts needed for clinical treatment and this may change their biological characteristics and result in cell transformation. There is also a risk of microbial contamination during in vitro artificial cell expansion of MSCs. Thus, MSC engraftment requires rigorous bacterial testing, as well as control of the time and dose. Moreover, whether MSCs are traditional ‘stem cells’ remains controversial. For example, both MSCs and fibroblasts have a similar appearance, surface markers, and differentiation ability. Furthermore, lacking in homogeneity and comparability, current clinical trials on MSC transplantation do not ensure stable and reproducible clinical efficacy. The long-term safety of MSC therapy is also not supported by data from large-scale, clinical, double-blind, randomized and controlled trials.

## Conclusion

Since its first long-term success in 1954, kidney transplantation has represented the best treatment for patients with ESRD. Immunosuppressive drugs taken by patients after transplantation heighten the risk of opportunistic infections and organ toxicity, which can affect the quality of life of patients as well as graft survival. MSCs have recently emerged as a prominent candidate for cell-based therapies for GVHD [4] and kidney transplantation [22, 27], and in many other clinical areas. Four main characteristics predetermine the clinical applications of MSCs: (1) homing to damaged tissues and inflammatory sites; (2) differentiation into various cell types and tissues; (3) secretion of bioactive molecules; (4) immunomodulation by their immunosuppressive and anti-inflammation properties. The efficacy of MSC therapy depends on the relative amounts of proinflammatory and anti-inflammatory cytokines, and pretreatments with proinflammatory cytokines (such as IFN-γ) improve the efficacy of MSC-based therapy [11]. Therefore, to optimize therapy, feasible approaches and relevant biomarkers are needed so that the inflammatory status of patients at the time of MSC infusion can be monitored.

Numerous preclinical trials have shown that MSCs effectively improve outcome after kidney transplantation, but their value is limited due to the different mechanisms of action of human and murine MSCs. Although many clinical trials have demonstrated promising outcomes of MSC-based therapy, the best source of MSCs, the optimal timing, dosage, route and frequency of MSC administration, as well as long-term post-transplantation safety remain unclear. Nevertheless, we expect that MSCs will contribute substantially to the success of human kidney transplantation, while large-scale, multi-center clinical trials are needed to further validate their clinical effects.

## Abbreviations

ADMSC: Adipose-derived mesenchymal stem cell
BM-MSC: Bone marrow-derived mesenchymal stem cell
CsA: Cyclosporine A
ESRD: End-stage renal disease
GVHD: Graft-versus-host disease
HLA: Human leukocyte antigen
IDO: Indoleamine 2,3-dioxygenase
IFN: Interferon
IL: Interleukin
MHC: Major histocompatibility complex
MSC: Mesenchymal stem cell
TLR: Toll-like receptor
